# Supplementary material for: Research ethics and collaborative research in health and social care: Analysis of UK research ethics policies, scoping review of the literature, and focus group study
Source: PLoS One. 2023 Dec 22;18(12):e0296223. doi: 10.1371/journal.pone.0296223 (PMC10745183; doi:10.1371/journal.pone.0296223)
Supplement: S1 File — (DOCX) [file pone.0296223.s001.docx]

# Scoping review of the literature – Search strings

| **Database** | **Search criteria** |
| --- | --- |
| **Web of Science** | TITLE: ("research ethics" or "research approval*" or "research governance approval*" or "ethic* review" or "research ethics committee*" or "institutional review board*") *AND* TITLE: ("co-creation"  OR "co-production"  OR "co-design"  OR "participatory research"  OR "participatory action research"  OR "community-based participatory research"  OR "community based participatory research"OR "implementation science"  OR "improvement science" or "improvement"  OR "implementation research"  OR "evaluation" OR "collaborat* research" or "qualitative research")  Timespan: 2010-2020. Databases:  WOS, BCI, KJD, MEDLINE, RSCI, SCIELO.  Search language=English |
| **PubMed** | ("research ethics"[Title] OR "research approval*"[Title] OR "research governance approval*"[Title] OR "ethic* review"[Title] OR "research ethics committee*"[Title] OR "institutional review board*"[Title]) AND ("co-creation" [Title] OR "co-production" [Title] OR "co-design" [Title] OR "participatory research" [Title] OR "participatory action research" [Title] OR "community-based participatory research" [Title] OR "community based participatory research"OR "implementation science" [Title] OR "improvement science"[Title] OR "improvement" [Title] OR "implementation research" [Title] OR "evaluation"[Title] OR ("collaborat* research"[Title] OR "qualitative research"[Title])  Timespan: 2010-2020.  English |
| **PsycInfo** | Search terms used:  co-creation  co-design  co-production  community  based  participatory  research  community-based  ethic*  review*  evaluation  implementation  science  improvement  collaborat*  qualitative  institutional  review  board*  action  approval*  ethics  committee*  governance  approval  limit 6 to (english language and yr="2010 - 2020") |
