# Supplementary material for: Research ethics and collaborative research in health and social care: Analysis of UK research ethics policies, scoping review of the literature, and focus group study
Source: PLoS One. 2023 Dec 22;18(12):e0296223. doi: 10.1371/journal.pone.0296223 (PMC10745183; doi:10.1371/journal.pone.0296223)
Supplement: Attachment — Submitted filename: Response to reviewers FINAL.docx [file pone.0296223.s003.docx]

Chiara De Poli

Care Policy and Evaluation Centre

London School of Economics and Political Science

Houghton Street, London, WC2A 2AE, UK

23^rd^ November 2023

Dr Molina Pérez

We are grateful to you and the reviewers for the time and constructive feedback on our manuscript “Research ethics and collaborative research in health and social care: analysis of UK research ethics policies, scoping review of the literature and focus group study” (Manuscript ID PONE-D-23-23653).

We have incorporated the suggestions we have received and we wish to submit a revised version for further consideration in the journal.

Changes in the initial version of the article are track-changed in the revised version. We also provide a point-by-point response explaining how we have addressed each of the reviewers’ comments.

In response to an editorial comment, we provide here the updated Competing Interests section:

*Chiara De Poli had research grant funding from the National Institute for Health and Care Research, School of Social Care Research, grant number 106152/CBF/LSECDP-IF14. Currently, as a co-applicant, she has research grant funding by the National Institute of Health and Care Research - Three Schools Dementia Programme, grant number 102645/3SDRP/LSEACH-DP05.*

*Jan Oyebode currently has research grant funding from the National Institute for Health Research, grant number 204266, Social Care for People with Young Onset Dementia.*

We confirm that this does not alter our adherence to  PLOS ONE policies on sharing data and materials.

Thank you for the opportunity to revise our manuscript.

Yours sincerely,

Chiara De Poli

Jan Oyebode

**Journal requirements**

*When submitting your revision, we need you to address these additional requirements.*

1. *Please ensure that your manuscript meets PLOS ONE's style requirements, including those for file naming. The PLOS ONE style templates can be found at*

[*https://journals.plos.org/plosone/s/file?id=wjVg/PLOSOne_formatting_sample_main_body.pdf*](https://journals.plos.org/plosone/s/file?id=wjVg/PLOSOne_formatting_sample_main_body.pdf) *and*

[*https://journals.plos.org/plosone/s/file?id=ba62/PLOSOne_formatting_sample_title_authors_affiliations.pdf*](https://journals.plos.org/plosone/s/file?id=ba62/PLOSOne_formatting_sample_title_authors_affiliations.pdf)

We have reviewed the journal style templates and revised as needed.

2. *Thank you for stating the following in the Competing Interests section:*

*“Chiara De Poli had research grant funding from the National Institute for Health Research, School of Social Care Research, grant number 106152/CBF/LSECDP-IF14.*

*Jan Oyebode currently has research grant funding from the National Institute for Health Research, grant number 204266, Social Care for People with Young Onset Dementia.”*

*Please confirm that this does not alter your adherence to all PLOS ONE policies on sharing data and materials, by including the following statement: "This does not alter our adherence to  PLOS ONE policies on sharing data and materials.” (as detailed online in our guide for authors* [*http://journals.plos.org/plosone/s/competing-interests*](http://journals.plos.org/plosone/s/competing-interests)*).  If there are restrictions on sharing of data and/or materials, please state these. Please note that we cannot proceed with consideration of your article until this information has been declared.*

*Please include your updated Competing Interests statement in your cover letter; we will change the online submission form on your behalf.*

We have included the updated Competing Interests statement in our cover letter.

3. *Please review your reference list to ensure that it is complete and correct. If you have cited papers that have been retracted, please include the rationale for doing so in the manuscript text, or remove these references and replace them with relevant current references. Any changes to the reference list should be mentioned in the rebuttal letter that accompanies your revised manuscript. If you need to cite a retracted article, indicate the article’s retracted status in the References list and also include a citation and full reference for the retraction notice.*

We have updated the reference list and removed double entries for the same publication.

**Reviewer #1**

*The study is designed to address whether the current ethics policies, guidelines and processes are fit for purpose in the context of “collaborative research” in health and social care, but from my perspective there are some key issues:*

1. *The authors have reviewed and analysed only the UK research ethics policies, including for social sciences, and state that “…. the UK system draws on international standards and governance mechanisms established by the Nuremberg Code and the Declaration of Helsinki, shares this bedrock with other countries, and in this sense it is an example of a modern research ethics system.	However, excellent guidance documents in this area not only from UK itself, (*[*https://www.publicengagement.ac.uk/sites/default/files/publication/cbpr_ethics_guide_web_november_2012.pdf*](https://www.publicengagement.ac.uk/sites/default/files/publication/cbpr_ethics_guide_web_november_2012.pdf) *) but also from international organizations (* [*https://www.who.int/publications/i/item/9789241502948*](https://www.who.int/publications/i/item/9789241502948)*;* [*https://www.who.int/publications/i/item/9789241505475*](https://www.who.int/publications/i/item/9789241505475)*;* [*https://ahpsr.who.int/publications/i/item/2019-12-02-ethical-considerations-for-health-policy-and-systems-research*](https://ahpsr.who.int/publications/i/item/2019-12-02-ethical-considerations-for-health-policy-and-systems-research) *and* [*https://equinetafrica.org/sites/default/files/uploads/documents/PAR_Methods_Reader2014_for_web.pdf*](https://equinetafrica.org/sites/default/files/uploads/documents/PAR_Methods_Reader2014_for_web.pdf)*) already exist but have not been included nor alluded to. The “Methods Reader” on Participatory Action Research in health systems published in 2014 in collaboration with WHO* [*https://equinetafrica.org/sites/default/files/uploads/documents/PAR_Methods_Reader2014_for_web.pdf*](https://equinetafrica.org/sites/default/files/uploads/documents/PAR_Methods_Reader2014_for_web.pdf) *discusses the ethical challenges in participatory research very thoughtfully (including many of the issues identified by the authors of this paper) and provides a list of ethical principles relevant for this type of research.*

*The question to be additionally answered perhaps is why the UK HRA has not embedded the existing guidance including international ethics policies and guidance relevant for participatory action research in its own guidance framework? In any case, it would be relevant to review these additional documents in the paper, and acknowledge the existence of excellent guidance and principles that already exist.*

Thank you for this comment. The starting point of our work was the policy and regulatory framework used in the UK to oversee research that involves human participants (Health Research Authority, 2017). The UK framework (para 3.4) states that it reflects relevant domestic legislation (see Appendix 2 of the UK policy framework) and draws on relevant sources such as publications about good research practice (including the Declaration of Helsinki), but “*cannot exhaustively compile all the principles, requirements and standards that may be issued separately by individual bodies with an interest in research*”. In outlining the scope of the policy framework, para 3.5 also adds that “*the principles and responsibilities set out in this policy framework are deliberately at a high level and apply to health research and social care research equally. However, health research and social care research can be different in nature, scale, setting, volume and funding, as well as in the mix of stakeholders, organisational context and range of academic disciplines. Individuals and organisations with responsibilities under this policy framework are therefore expected to take appropriate account of these differences when reflecting the principles and responsibilities set out in this document in their own policies, procedures and practice*.”

Hence, in the view of the HRA, the policy framework provides the overarching principles of good practice in the management and conduct of health and social care research that take account of legal requirements and other standards. This would appear to be why existing guidance, including international ethics policies and guidance relevant for participatory and collaborative research, has not been embedded in the UK framework. The principles and processes outlined in some of the guidance documents that you kindly shared with us are (or could be) adopted and reflected in the policies, procedures and practice of individuals and organisations with responsibilities *under* the policy framework, rather than in the policy framework itself.

We have revised the Introduction to clarify the starting point of our work (lines 158-164). In the Discussion we have added the contribution of various organisations to providing relevant guidance that can be used to address the requirements of research ethics policy and regulations in a meaningful way (lines 751-755).

2. *The scoping review was designed to respond to specific questions*

- *How does the current research ethics system work for collaborative research in health and social care?*
- *What are the challenges that the current research ethics framework poses to collaborative research in health and social care?*
- *What options have been discussed in the literature to overcome these challenges?

  It was not designed to answer the question – does the application of the current research ethics framework by ethics committees actually ‘protect’ (the interests of) research participants?
  Similarly, the FGD was designed to respond to challenges in obtaining ethics approval, and how the approval processes could be better suited to “collaborative” research. The responses of the FGD participants mostly suggest ways that the ethics committees response could have been more tailored to the type of (collaborative) research. The FGD does not respond to the question of whether interventions made by ethics committee actually protect the interests of the research participants.
  Thus the introductory paragraph starting line 58, while perhaps relevant in and of itself, and the statement that (lines 633-637) “……. this work does not identify any instance in which research ethics had been acknowledged to have a positive impact on research nor did we find evidence of whether and why research ethics achieves its objectives….” appear inappropriate for this paper and not justified either by the objectives nor the results of the study. I would suggest deleting these sections.
  Here I do not deny the existence of ethics creep nor do I deny the absence of evidence for whether ethics committees actually do what they are supposed to do; the absence of evidence however (to us a clichéd term) is not evidence of absence. Nor does it mean that academicians have not tried to measure the actual effectiveness of ethics review (i.e. that the interests of research participants and communities were protected). Some of these challenges have been very thoughtfully described in the recent article by Lynch et al (*[*https://www.sciencedirect.com/science/article/abs/pii/S0277953621009461*](https://www.sciencedirect.com/science/article/abs/pii/S0277953621009461)*)*

Thanks for this comment about the effectiveness of research ethics review.

The Introduction of the article aims to position our work in the context of the ongoing debate about research ethics processes. We think it is important to acknowledge that the research on research ethics so far has been focusing mainly on what we can measure (e.g., structures and processes) as a proxy for effectiveness, so our understanding of the effectiveness of such structures and processes is still limited. However, our own focus is, as you accurately state, on how well current ethics processes support participatory and collaborative research rather than on effectiveness of protection for participants per se. We would therefore suggest to keep the introductory paragraph starting line 74 but have rephrased the initial part of the paragraph to take your review comment into account.

In relation to your comment on our remarks in the discussion around line 633 about a lack of findings on positive impacts of ethics processes, we take your point. The scoping review was not conducted to map evidence on the effectiveness of research ethics per se, although our analytical framework did allow for a residual theme “Other” to identify additional topics beyond the a priori themes. Under this additional theme, we did not identify any evidence of effectiveness. In relation to the focus group, participants could have shared experiences of research ethics processes ‘working well’ given the exploratory nature and the broad scope of the discussion. However, admittedly we did not explicitly solicit comments about this.

Hence, we agree that we do not have any evidence to support the claim about whether and why research ethics achieves its objectives. We have therefore revised this aspect of the discussion and have simply underscored the silence of the reviewed literature on this aspect (see lines 689-695).

3. *I am a little confused why the authors have chosen to use the term “collaborative” research and not the more widely used “participatory” research. Collaborative research is more generally used for research that is done collaboratively with other researchers (researchers from high income countries working collaboratively with researchers from low and middle income countries, researchers from different sectors (health and agriculture for example) working collaboratively etc.). Actually there are research ethics guidelines for collaborative research of this type (cf* [*https://cioms.ch/wp-content/uploads/2016/08/International_Ethical_Guidelines_for_Biomedical_Research_Involving_Human_Subjects.pdf*](https://cioms.ch/wp-content/uploads/2016/08/International_Ethical_Guidelines_for_Biomedical_Research_Involving_Human_Subjects.pdf)*)*

Thanks for this comment. We are aware that terminology is often not used consistently and also seems to vary across disciplines and settings. To some extent our scoping review provides evidence of this as well.

We had extensive discussion about this within the research team. We settled on using ‘collaborative’ as shorthand for ‘participatory and collaborative research’, an umbrella term for various research approaches (e.g., participatory action research, community-based participatory research) and collaborative designs (e.g., co-creation, co-design, co-production) where participants are actively involved in shaping the research. We refer to collaborative research as research that moves away from the traditional divide between knowledge producers (typically academic researchers) and knowledge users (such as policy-makers, service providers, or the public) and from the primacy of academic knowledge against other types of knowledge (e.g., tacit knowledge or lived experience).

In the UK context, the term participatory is often associated with Participatory Action Research, which is just one type of collaborative research. This contributed to our preference for not using it.

We have revised the Introduction (lines 135-142) to justify our choice of terminology.

4. *Much of the literature that is cited is from before 2015 and hopefully much progress has been made in the last 8-10 years. Most of the articles included in the scoping review are a critical reflection on ethical issues. The authors may wish to comment, based on the reflections, if anything has changed in the past decade or not.*

Thanks for this comment. We have reflected on the trend over time of the literature that we identified with the scoping review. There seem to be the following issues at play:

- The scope of the literature review was at the intersection of research ethics and collaborative research (as defined above). Neither area was easy to pin down, terminology was not used consistently within or across disciplines, countries, and research traditions. This may reflect in how effective the search strings used in the literature review were. Indeed, we acknowledged this as a potential limitation of our work.
- It seems to us that if, on the one hand, the academic literature has not grown substantially, on the other there seems to be a growing interest in this topic in the grey literature. We cannot provide definite evidence of this because the grey literature was outside the scope of the literature review. However, we are aware that methodological guidance and good practice advice have become increasingly available from various sources, e.g., organisations under the WHO umbrella (as you suggested), organisations of national research infrastructures, Universities that provide research ethics oversight for the research they sponsor, and organisations acting as research facilitators and gatekeepers.
- It seems that the peak of articles published in the early to mid-2010s may coincide with a large wave of research studies using collaborative approaches. In that period researchers started sharing their experiences of navigating the research ethics approval process for collaborative studies, reflecting on the tensions between research ethics and their research approaches and often providing guidance and advice to others dealing with the same issues. This may have helped develop a community that contributed to normalise research ethics practices which were unusual in that initial phase. As a result, the scholarly interest in writing about this topic in academic outlets has possibly faded away, to be replaced by methodological guidance and good practices available in the grey literature.

We have revised the Discussion of the article accordingly (see lines 684-697).

5. *Minor point – in line 93, the authors have defined justice rather narrowly in terms of who gets the benefits, whereas research ethics guidance talk about fairness in terms of who gets to participate, who bears the brunt of the risks, fairness of research partnerships and so on. Perhaps the authors may wish to define justice more broadly?*

We have revised the definition of justice more broadly, as per Council for International Organizations of Medical Science (2002) (see lines 114-116).

6. *Cf line 396 – Membership and training of REC members are covered both in the CIOMS and the WHO guidelines.*

Thanks for raising this point, we have edited the Discussion to acknowledge the difference between the UK policy and the WHO guidelines (see lines 676-677).

**Reviewer #2**

*This article is clearly documented and its methodology is robust. The topic is essential and perfectly analyzed. It may have implications for international contexts beyond UK. Several proposals to improve the Ethics process such as specific RECs and training about the different types of research could apply abroad and be rather simple to implement. The point of participants' data protection (GDPR) may be more underlined as a barrier to RECs decisions in some countries (beyond anonymity or consent). It does not seem to be such a difficulty in the UK according to this article.*

Many thanks for your feedback.

**Cited references**

Council for International Organizations of Medical Science (WHO). (2002). *International ethical guidelines for biomedical research involving human subjects*. CIOMS. https://cioms.ch/publications/product/international-ethical-guidelines-for-biomedical-research-involving-human-subjects-2/

Health Research Authority. (2017). *UK Policy Framework for Health and Social Care Research*. https://www.hra.nhs.uk/planning-and-improving-research/policies-standards-legislation/uk-policy-framework-health-social-care-research/
